# Tools and Methods for Achieving Wi-Fi Sensing in Embedded Devices

**DOI:** 10.3390/s25196220

**Published:** 2025-10-08

**Authors:** Jesus A. Armenta-Garcia, Felix F. Gonzalez-Navarro, Jesus Caro-Gutierrez, Conrado I. Garcia-Reyes

**Affiliations:** Engineering Institute, Universidad Autonoma de Baja California, Calle de la Normal S/N Col. Insurgentes Este, Mexicali 21100, Mexico; albany.armenta@uabc.edu.mx (J.A.A.-G.); jesus.caro@uabc.edu.mx (J.C.-G.); conrado.ivan.garcia.reyes@uabc.edu.mx (C.I.G.-R.)

**Keywords:** Wi-Fi sensing, HAR, deep learning, data augmentation

## Abstract

Wi-Fi sensing has emerged as a powerful approach to Human Activity Recognition (HAR) by utilizing Channel State Information (CSI). However, current implementations face two significant challenges: reliance on firmware-modified hardware for CSI collection and dependence on GPU/cloud-based deep learning models for inference. To address these limitations, we propose a two-fold embedded solution: a novel CSI collection tool built on low-cost microcontrollers that surpass existing embedded alternatives in packet rate efficiency under standard baud rate conditions and an optimized DenseNet-based HAR model deployable on resource-constrained edge devices without cloud dependency. In addition, a new HAR dataset is presented. To deal with the scarcity of training data, an Empirical Mode Decomposition (EMD)-based data augmentation method is presented. With this strategy, it was possible to enhance model accuracy from 59.91% to 97.55%. Leveraging this enhanced dataset, a compact DenseNet variant is presented. An accuracy of 92.43% at 232 ms inference latency is achieved when implemented on an ESP32-S3 microcontroller. Using as little as 127 kB of memory, the proposed model offers acceptable performance in terms of accuracy and privacy-preserving HAR at the edge; it also represents a scalable and low-cost Wi-Fi sensing solution.

## 1. Introduction

Wi-Fi is a communication technology that is currently present in a myriad of devices. While its primary purpose is to provide mechanisms for connecting devices to a network, it is possible to take advantage of the channel estimations made by Wi-Fi devices for human sensing applications, giving rise to what is known as Wi-Fi sensing. Wi-Fi sensing involves collecting, analyzing, and processing either Received Signal Strength Indicator (RSSI) or Channel State Information (CSI). However, CSI has proven to be more effective for capturing fine-grained movements related to human motion if compared to RSSI as it contains information of multipath effects such as scattering, fading, and path loss [1]. By having access to this measurement, it is possible to develop human activity recognition, gesture recognition, and breathing rate monitoring applications [2,3,4,5]. Unlike other wireless sensing technologies such as camera-based systems, Wi-Fi sensing preserves privacy by avoiding visual recordings. Its ubiquity in routers, IoT devices, and smartphones, combined with the fact that it does not require line-of-sight (LoS) with the person being monitored [6], significantly enhances its privacy benefits. These reasons make Wi-Fi sensing a scalable solution for smart homes, healthcare monitoring, and industrial safety.

Wi-Fi CSI signals originate in the physical layer (PHY) and their purpose is to mitigate the multipath fading effects of a wireless communication channel. This allows Multiple-Input–Multiple-Output (MIMO)-based systems to adapt to current channel conditions and thereby optimize aspects such as beamforming, power allocation, and modulation schemes.

In a MIMO system, i.e., an arragement of *n* receiver antennas and *m* transmitter antennas, the received signal can be expressed as a signal vector *Y* of size *n* defined in a time *t* as

(1) $$\begin{array}{c} Y_{t}=HX_{t}+\eta_{t} \end{array}$$

where *H* is a complex matrix of dimension n×m representing the wireless channel known as CSI and $X_{t}$ is the transmitted signal vector of size *m* plus a noise vector $\eta_{t}$. Thus, the system estimates *H*, which contains information about the multipath fading effects in the channel, based on knowledge of $Y_{t}$ and $X_{t}$. Knowing this, the system adapts to current channel conditions, enhancing as a result the communication process [7].

**Figure 1:**
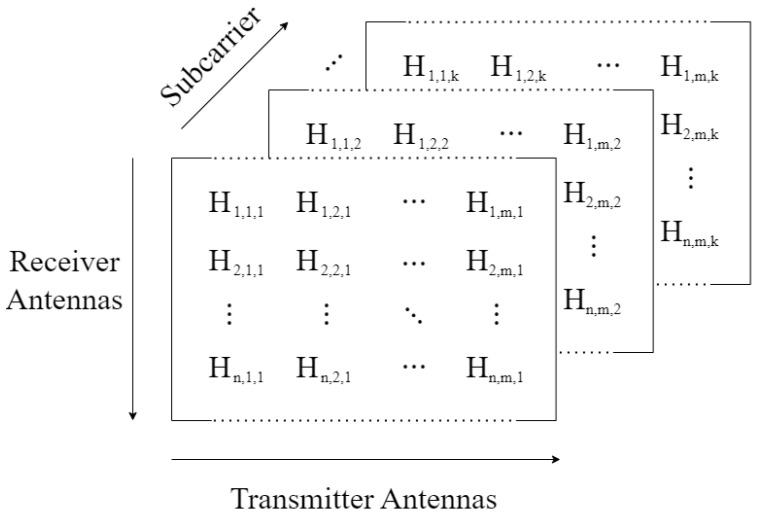
CSI matrix representation.

CSI is estimated based on predefined symbols known as Long Training Field (LTF) symbols, which are sent by the transmitter in each PHY frame preamble. As Wi-Fi transmits using Orthogonal Frequency Division Multiplexing (OFDM), the transmission of the signal is achieved by subcarriers that are orthogonal to each other. Thus, LTFs are transmitted across all subcarriers, resulting in *H* being a collection of matrixes, where each matrix corresponds to one subcarrier [8], as depicted in Figure 1. Each matrix element of *H* for a subcarrier *f* for a stream between antenna *a* and antenna *b* for a time *t* is given by

(2) $$\begin{array}{c} H_{a,b,f}\left(t\right)=\alpha_{a,b,f}\left(f\right)e^{-j\theta_{a,b,f}\left(t\right)} \end{array}$$

where $\alpha_{a,b}$ is the amplitude that represents attenuation and $e^{-j\theta_{a,b}\left(f\right)}$ the phase shift due to multipath propagation.

In the Wi-Fi 802.11n standard [9], a 20 MHz channel consists of 64 subcarriers, which are divided into 52 user data subcarriers, four pilot subcarriers used for synchronization and correction, and eight null subcarriers that are used as guard bands for adjacent channels, each with a spacing of 312.5 kHz [10].

Most Wi-Fi sensing system proposals rely on the use of deep learning models, whose inference relies on the use of computers with GPUs or even cloud services [11]. These systems consume more energy than microcontrollers, which can hinder scalability, demand specialized hardware, and—when reliant on cloud services—require continuous connectivity to operate effectively. Furthermore, these systems rely on the use of specific legacy Wi-Fi devices that need custom firmware for enabling CSI collection, forcing interested researchers on the topic to resort to buying them from third parties or second-hand, also affecting the system’s scalability.

An alternative is to perform both model inference and CSI collection directly on embedded devices with integrated Wi-Fi capabilities, such as the ESP32 microcontroller. Such an approach mitigates the aforementioned dependencies. However, the deployment of sophisticated deep learning models on these platforms remains a significative challenge, primarily due to their constrained memory resources and limited computational power [11,12].

Given the detailed structure of CSI and its ability to capture fine-grained movements, leveraging this information for Human Activity Recognition (HAR) requires both data collection tools and processing methods that ensure reliable performance by addressing the challenges of inference on embedded devices. Hence, this work aims to address these needs by introducing a novel tool and methods for enhancing Wi-Fi sensing in embedded devices through deep learning and data augmentation. The main contributions of this work can be summarized as follows:

- A CSI collection tool named the ESP32 CSI Web Collecting Tool, which is an alternative that simplifies tool configuration and achieves packet rates of up to 180 packets per second. It supports USB-to-UART serial transmission through the ESP32 USB port, allowing for packet rates of up to 80, outperforming current state-of-the-art ESP32-based CSI collection tools working at standard baud rates.
- A dataset comprising over 1000 samples containing CSI measurements from five different activities, which can be used for training classification models.
- An adaptation of a data augmentation method based on Empirical Mode Decomposition (EMD) for generating meaningful synthetic samples used for training classification models. By using this method, it was possible to surpass the ten thousand samples that were used to train a deep learning model.
- The implementation of a HAR model on an embedded device with near-real-time response and presenting accuracies above 90% by using as input CSI that was collected using the same.

These contributions enhance the feasibility of Wi-Fi sensing using low-cost embedded devices, showing that reliable HAR can be achieved with near-real-time data processing, an essential milestone toward the scalable deployment of Wi-Fi-based sensing systems.

This paper is organized as follows: Section 2 reviews the state-of-the-art in Wi-Fi CSI-based sensing, beginning with the most commonly used CSI collection tools. It then examines traditional Wi-Fi sensing techniques for HAR and recent advancements enabled by embedded devices. Section 3 introduces the ESP32 CSI Web Collecting Tool, detailing its functionality, configurable parameters, and internal component interactions. Section 4 describes the adaptation of an EMD-based data augmentation method for generating synthetic samples to enhance HAR performance. Section 5 outlines the deep learning model architecture implemented for HAR. Section 6 explains the methodology for data collection, as well as the training, testing, and deployment of the model on an embedded device. Section 7 presents the experimental results and, finally, Section 8 concludes this paper.

## 2. Related Works

### 2.1. Wi-Fi Collecting Tools

Over the years, tools for collecting CSI from Wi-Fi devices have been developed since CSI cannot be obtained directly from these devices. As it is physical layer information, it is used for adapting communications according to channel estimations without the need to report CSI outside of the Wi-Fi device directly. Therefore, modifications to the firmware of specific Wi-Fi devices are necessary to collect CSI.

The most used tool reported in the literature for collecting CSI is the Linux 802.11n CSI Tool developed by Halperin et al. This tool applies a custom firmware to the Intel 5300 Network Card Interface (NIC) and uses a debug mode available for computers with the Ubuntu Linux kernel. This tool reports CSI from a group of 30 subcarriers for each 802.11n frame received with a signed 8-bit resolution for either a 20 MHz or 40 MHz channel [13].

On the other hand, the Atheros CSI Tool is available on various Atheros chipsets. It features a 10-bit resolution for measurements, enabling the reporting of up to 56 subcarriers for a 20 MHz channel and 114 for a 40 MHz channel. Unlike the previous one, which is only available for a range of kernel versions and a single network card, the Atheros CSI Tool is compatible with OpenWRT, which opens up a range of possibilities for working with embedded devices [14].

The Nexmon CSI Extractor tool allows one to collect CSI from either Broadcom or Cypress Wi-Fi, with support for the Raspberry PI platform and smartphones. It is a C-based firmware patching framework, compatible with the Wi-Fi 802.11a/n/ac/ax standards. This tool reports information from 256 subcarriers if working with the 802.11a/n/ac standards in an 80 MHz channel and from 2048 subcarriers under the 802.11ax standard in a 160 MHz channel [15,16].

As mentioned previously, these aforementioned tools require changes to the devices’ firmware. They are limited to specific network devices that may be considered legacy or that are discontinued, e.g., the Intel 5300 NIC used for the Linux 802.11n CSI Tool. This hinders the acquisition of these devices and limits the scalability of Wi-Fi sensing systems. An alternative is the use of ESP32 microcontrollers, with integrated Wi-Fi and Bluetooth, as it features a Wi-Fi API developed by Espressif that allows for collecting CSI from Wi-Fi 802.11n in 20 and 40 MHz channels. The ESP32 CSI tool utilizes this Wi-Fi API to collect CSI data from 64 subcarriers within a 20 MHz channel using ESP32 microcontrollers. It offers two operation modes: active and passive, with each CSI value encoded into ASCII format. In active mode, two ESP32s are required: one programmed as an active access point and the other as a station. This setup establishes a communication link between the two devices, from which CSI estimation is performed. In contrast, in passive mode, a single ESP32 is programmed as a sniffer that connects to any access point and collects CSI from all devices connected to the same access point and in the channel [17]. However, configuring this tool may require experience using the ESP32’s development framework, as well as modifications to its source code to achieve optimal performance.

A summary of these Wi-Fi CSI collecting tools is presented in Table 1.

To overcome the limitations of the ESP32 CSI tool, the proposed ESP32 CSI web collecting tool streamlines configuration by providing a web-based interface for setting communication and operational parameters directly in the field. It leverages the ESP32’s dual-core architecture and supports USB-to-UART serial transmission via the onboard USB port, SD card storage, and device-to-device UART communication through available GPIO pins. Additionally, CSI data can be reported in binary format, enabling the transmission of a higher volume of packets at standard baud rates.

### 2.2. Wi-Fi Sensing for HAR

Traditional Wi-Fi sensing relies on the use of one of the aforementioned CSI collecting tools running on a computer equipped with the corresponding NIC. For example, the authors of [18] used the Linux 802.11n CSI tool to collect CSI and construct spectrogram images from it. These were then processed by a ResNet model, based on Convolutional Neural Networks (CNNs), running on a personal computer, equipped with a dedicated GPU, to perform binary classification for fall and non-fall activities. With this approach, the authors achieved an accuracy of over 92%, while generating CSI spectrograms and classifying using the model took around 78.9 ms. Similarly, in [19], a CNN was used for extracting CSI features from CSI collected using the Atheros CSI tool running on a laptop. These features were classified by an ensemble classifier composed of a Random Forest, an SVM, and a Multiple-layer Perceptron. With this approach, an accuracy of 98.9% was achieved for identifying the activities of sit down, jump, wave, pick up, walk, and run. Moreover, in [6], a Long Short-Term Memory (LSTM)-based model was used to interpret temporal dependencies from CSI for recognizing activities such as walking, running, sitting, standing, and falling, as well as no activity in the sample. The Linux 802.11n CSI tool was used for collecting CSI from two laptops, achieving an overall accuracy of 95%. In [20], the use of a principal component-based wavelet CNN, i.e., a CNN that takes as input the second and third principal component extracted from CSI processed with a Savitzky–Golay filter, was explored for later combining the approximation coefficients obtained by the DWT from processed CSI with the feature maps obtained from the convolutional layers, obtaining an accuracy of 95% for recognizing 16 different activities using CSI collected from laptops and desktop computers running the Linux 802.11n CSI tool. By leveraging attention mechanisms and a ResNet-based architecture, the authors of [21] achieved in-domain and cross-domain recognition with mean accuracies of 99.71% and 94.73%, respectively, using CSI images from the CSI ratio of the Widar3 dataset [22]. The Widar3 dataset consists of 16 volunteers, 15 gestures, 15 locations, and five orientations in three different environments, being a constantly used dataset for the development and evaluation of Wi-Fi sensing preprocessing and processing techniques, as well as for cross-domain model evaluation [23,24]. Additionally, the authors of [22] proposed the body-coordinate velocity profile (BVP), which describes power distribution over different velocities at which body parts are involved, enhancing cross-domain recognition performance.

However, these CSI collecting tools used for the presented works require specific network devices that are discontinued. The acquisition and use of multiple laptops or desktop computers for collecting and processing CSI hinders the deployment of Wi-Fi sensing systems and, although the reported results are encouraging, the selected techniques and model architectures are not suitable for its deployment in hardware-limited devices, such as microcontrollers, with near-real-time functioning.

### 2.3. Embedded Wi-Fi Sensing Applications

When using the ESP32 CSI tool, it is common to send CSI to another device with more computational power, such as a computer, for processing and analysis. For example, in [2], the CSI collected was sent to a computer for filtering noise with a wavelet-based denoising technique, preserving high-frequency variations introduced by human activities to recognize four activities: empty room, walking, sitting, and standing. Consequently, using an ensemble model, this approach achieved a mean cross-validation accuracy of 83.39%. Similarly, the authors of [25] developed a system that sent CSI collected with ESP32 devices to a computer that processed the data through a Python script. This script applied a Fourier method for interpolation and downsampling, addressing the issue of unstable packet rates inherent in the tool. In addition, discrete wavelet transform and principal component analysis were applied to extract relevant information related to a person’s breathing for finding the breathing rate based on power spectral density analysis, obtaining a root mean square error of 1.04 breaths per minute with this approach.

Moreover, several works have used single-board computers (SBCs) to process CSI data and even for deep learning-based classification. For instance, the system presented in [3] involved collecting CSI using the ESP32 CSI tool, while measurements were sent to a Jetson Nano for processing and classification. By applying a Hampel identifier, a Savitzky–Golay filter for processing, and a CNN, the authors obtained an accuracy of 95.57% for recognizing the activities of raising the left leg, raising the right arm, and stretching out. Likewise, in [26], a system for physical rehabilitation tracking focused on recognizing hand-based exercises is presented. In this system, CSI collected with an ESP32 was sent to Raspberry Pi for processing, classification, and exercise counting using a deep learning model with a network architecture optimized for devices with limited memory, achieving an overall accuracy of 91.22% for recognizing finger and wrist movements.

However, to demonstrate the capabilities of ESP32 for not only collecting CSI but also processing it, the ESP32 CSI tool was modified in [5] to enable data processing and apnea detection based on breathing rate estimation directly on the ESP32. The device applied a Hampel identifier and a low-pass filter to reduce noise from CSI and performed linear interpolation to treat the unstable packet rate. Finally, a peak detection algorithm estimated breathing rate, achieving a mean absolute deviation of 2.7 breaths per minute.

In this work, a deep learning model was implemented on an ESP32 microcontroller for HAR, with CSI collected using the ESP32 CSI web collecting tool, keeping the collecting and processing in embedded devices, thereby setting the foundations for low-cost and scalable Wi-Fi sensing systems.

## 3. The ESP32 CSI Web Collecting Tool

The proposed ESP32 CSI web collecting tool takes advantage of Espressif’s Wi-Fi CSI API to enable CSI collection. It uses two ESP32 development boards: a receiver (Rx) configured as an Access Point (AP) and a transmitter (Tx) that connects to the AP and transmits User Datagram Protocol (UDP) packets at a configurable rate. The Rx device estimates and reports CSI based on the tool’s configuration.

Upon initial boot after flashing the firmware, the ESP32 enters Configuration Mode, enabling an AP for setting operational parameters and selecting the device’s role (either Rx or Tx). The configurable parameters differ depending on the selected mode. In Rx, it is possible to configure the following parameters:

- MAC address: MAC address of the Tx device.
- Packet rate: The rate at which UDP packets are received.
- Wi-Fi channel: Wi-Fi channel. The channel must match the one selected in Tx.
- Sample format: Format in which the data will be sent or saved. It can be in either ASCII or binary format.
- Message structure: Measurements to be added into the message sent or saved data in the selected format. Values such as RSSI, timestamp, and antenna index, among others, can accompany CSI.
- Informer mode: Specifying if measurements will be sent through UART serial communication I/O pins (Serial Communication Mode), through the USB port to be seen in the ESP-IDF terminal, or by another program monitoring the USB port (Console Mode) or saved in an SD file (SD Mode).
- GPIO Pin Configuration (SD Mode): Define MISO, MOSI, CLK, and CS pins for SD card interfacing.
- GPIO Pin Configuration (Serial Mode): Tx, Rx pins, and baud rate for UART communication through GPIO pins.

Meanwhile, in Tx mode, the configurable parameters are only three, and their values, except for the MAC address, must match the values set in the Rx device.

- MAC address: MAC address of the Rx device.
- Packet rate: The rate at which UDP packets are sent to Rx.
- Wi-Fi channel: Wi-Fi channel. The channel must match the one selected in Rx.

**Figure A1:**
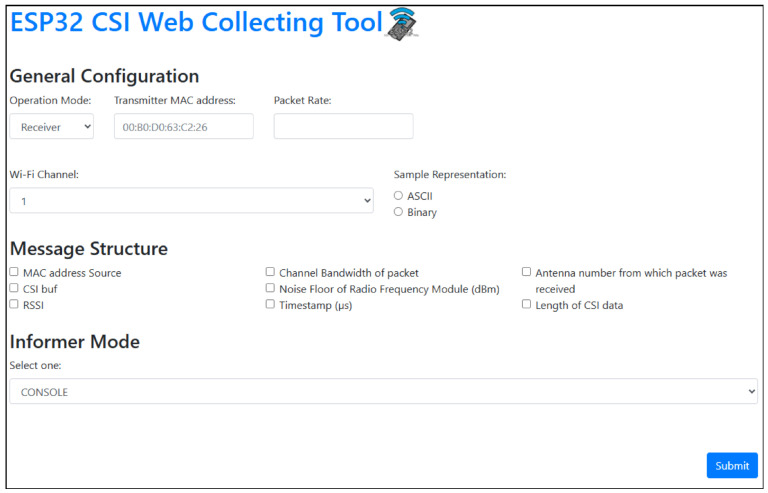
Web form for configuring the device as a receiver.

**Figure A2:**
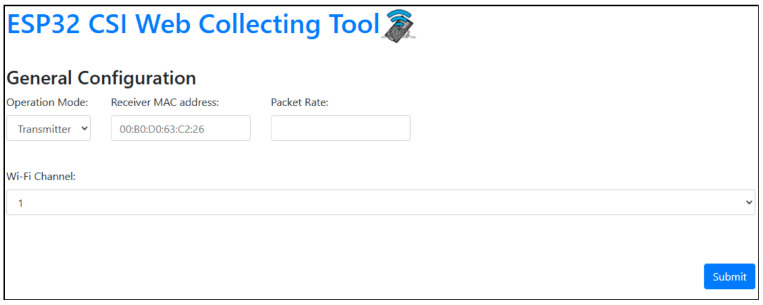
Web form for configuring the device as a transmitter.

The configuration web forms for Rx and Tx modes are illustrated in Figure A1 and Figure A2, respectively.

Upon submission of the web form, the specified configuration is saved to Non-Volatile Storage (NVS), allowing the device to retain its settings across reboots. The ESP32 will boot using the last stored configuration unless a manual reset is triggered via the board’s reset button, which clears all tool data and forces the device to return to Configuration Mode.

**Figure 2:**
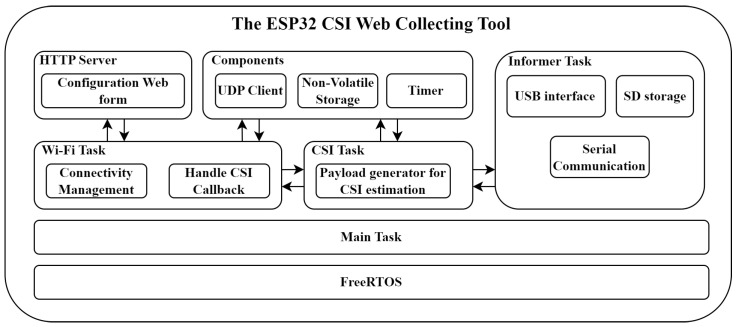
Overview of the ESP32 CSI web collecting tool architecture.

The ESP32 CSI web collecting tool is built on FreeRTOS, a real-time operating system optimized for microcontrollers that enables concurrent execution through task-based scheduling. By utilizing the ESP32’s dual-core architecture, the tool efficiently manages four primary tasks using FreeRTOS’s priority-based scheduling algorithm. Figure 2 illustrates the architecture of the proposed tool, showing the tasks and components involved in its operation.

### 3.1. The Wi-Fi Task

The Wi-Fi task is responsible for managing the device’s Wi-Fi connectivity, allowing the device to switch between Configuration Mode and CSI collecting. Upon device startup, it checks for an existing configuration stored in the NVS; if none is found, it starts an HTTP Server and enters Configuration Mode, enabling tool configuration through a web form. The defined configuration is then stored in the NVS.

Once configured, the device initiates according to the operation mode defined and starts the CSI task.

### 3.2. The HTTP Server

The configuration web form is handled through the HTTP Server, which registers Uniform Resource Identifiers (URIs) for fetching server resources, e.g., the web page and scripts for handling HTTP methods.

**Figure 3:**
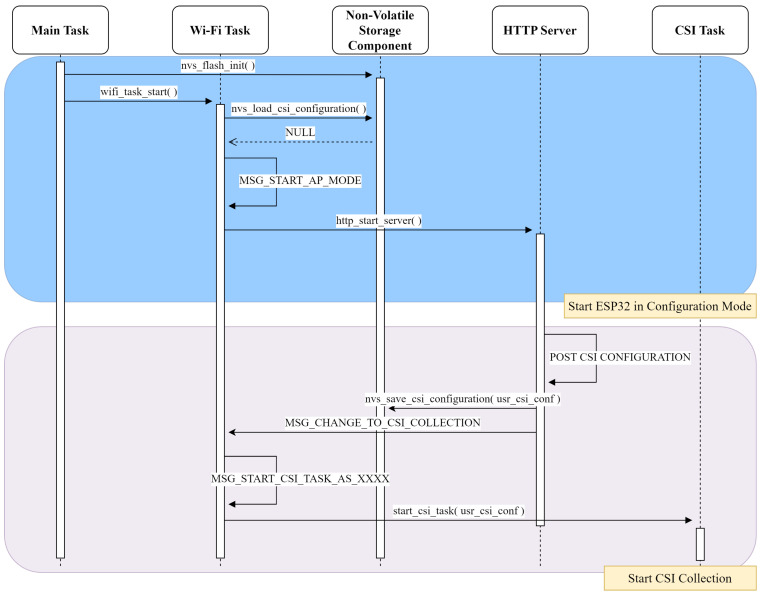
Sequence diagram for explaining the process of device starting in Configuration Mode and changing its operation mode to CSI collection.

To provide a detailed understanding of the interactions between the Wi-Fi task and the HTTP Server, Figure 3 presents a sequence diagram that outlines how these tasks communicate with each other through function calls, return values, and message passing using FreeRTOS task communication queues.

### 3.3. The CSI Task

**Figure 4:**
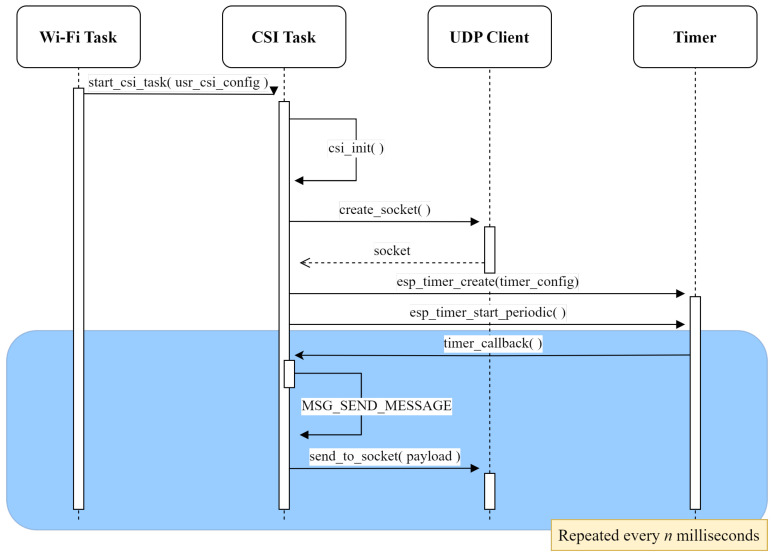
Sequence diagram for explaining the process of transmitting UDP packets for generating CSI.

The CSI task is initiated by the Wi-Fi task once the configuration is completed. Its operation depends entirely on whether the device was set as Tx or Rx. As Tx, the task creates a socket and uses a timer to send UDP packets to the Rx at a specified rate, triggering CSI estimations for every received packet. The interactions between the CSI task set in this mode and other tool tasks and components are illustrated in Figure 4.

**Figure 5:**
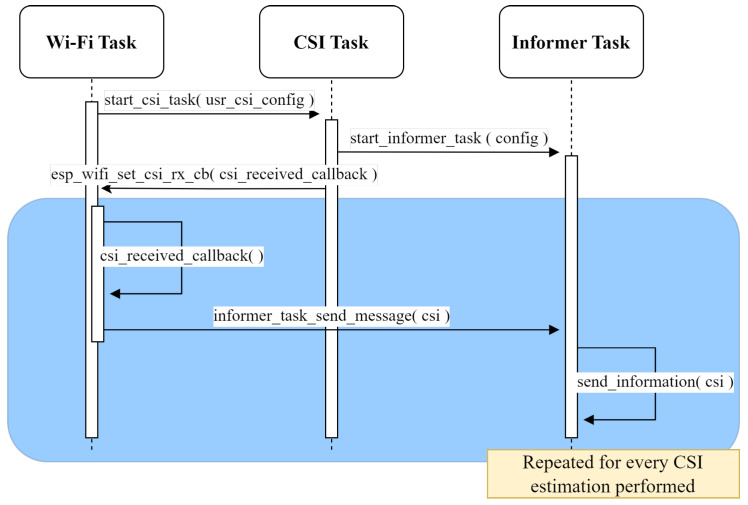
Sequence diagram for explaining the process of reporting estimated CSI from UDP packets received.

On the other hand, for Rx, the task defines a callback function for the Wi-Fi task that is executed upon each received packet after CSI estimation has been performed. Once the callback function is defined, it becomes idle after starting the informer task (see Figure 5).

### 3.4. The Informer Task

The informer task is triggered by the CSI task when the device is configured as Rx. Based on the informer mode selected via the web interface, the task either mounts an SD card to store CSI data or configures the UART for asynchronous transmission using the board’s GPIO pins or USB interface. The USB interface requires no additional setup as it operates using the framework’s default configuration.

## 4. EMD-Based Data Augmentation Method

### 4.1. Empirical Mode Decomposition

An Intrinsic Mode Function (IMF) is a concept from signal processing that is designed to analyze nonlinear and nonstationary time series data. By definition it must satisfy two conditions: the number of extrema and zero crossings must be either equal or differ at most by one and the mean value of the envelopes defined by the local maxima and minima must be zero. A full description of the frequency content of a non-linear and non-stationary signal such as CSI [27] can be obtained by decomposing it into its IMF components, where decomposition can be achieved by EMD [28].

To extract IMFs from a signal x(t) using EMD, a sifting process is employed. This procedure begins by identifying the local maxima and minima of the signal and connecting them through interpolation, thereby generating the upper and lower envelopes, respectively. The mean of these envelopes is then subtracted from the original signal, yielding the first component $h_{1}$:

(3) $$\begin{array}{c} h_{1}=x\left(t\right)-m_{1} \end{array}$$

The sifting process must be performed as long as $h_{1}$ fulfills the two conditions described above, turning finally into an IMF, namely, IMF $c_{1}$.

The IMF $c_{1}$ can be subtracted from x(t), obtaining the residue $r_{1}$. $r_{1}$ might still contain information of longer period components. This process leads to considering $r_{1}$ as a new reference signal that can be subjected to the sifting process. This procedure is repeated on every subsequent residue until the component $c_{n}$ or the residue $r_{n}$ becomes very small or when $r_{n}$ becomes a so-called monotonic function from which no more IMFs can be extracted. When this point is reached, it is said that decomposition of x(t) has been completed by obtaining *n* modes and a residue $r_{n}$. Hence, x(t) can be defined as

(4) $$\begin{array}{c} x\left(t\right)=\sum_{i=1}^{n}c_{i}+r_{n} \end{array}$$

### 4.2. The EMD-Based Data Augmentation Method

The method employed for synthetic subcarrier generation via EMD is adapted from the approach proposed by [29]. In order to explain the EMD adaptation, let us suppose that we have a dataset of CSI amplitudes H with *N* samples from the same individual. Each sample $H_{i}$ is a tensor with 64 subcarriers and 850 timesteps, i.e., a tensor of dimension 64×850. EMD is applied to each subcarrier $sc_{k}$ of $H_{i}$ for finding its IMFs. For each $sc_{k}$, seven IMFs plus a residue are extracted by EMD given that no less than seven IMFs are obtained by the sifting process from the samples. This gives as a result *N* tensors S of dimensions 64×8×850, which contain 850 steps for each IMF and residue for each $sc_{k}$ of sample $H_{n}$.

Following the same example, the *n* tensors S are further concatenated for constructing the tensor M of dimension N×64×8×850, i.e., the total number of samples × the number of subcarriers (64) × the number of IMFs extracted for each subcarrier (7+residue) × the timesteps. This structure allows the creation of synthetic samples by adding the modes taken from a pair of tensors A and B—which are part of M—from a same subcarrier index *k* in an alternating manner. This process preserves subcarrier-specific fading and as a result guarantees that the wireless channel will also be replicated in the synthetic samples for the same activity and individual. Each synthetic sample ${\hat{H}}_{i}$ can be seen as a group of 64 synthetic subcarriers, each being the result of the combination of modes of a pair:

(5) $$\begin{array}{c} synth\_sc_{k}=A_{\left(k,1,:\right)}+B_{\left(k,2,:\right)}+\cdots +A_{\left(k,8,:\right)}+B_{\left(k,8,:\right)} \end{array}$$

(6) $$\begin{array}{c} {\hat{H}}_{i}=\left[synth\_sc_{1},synth\_sc_{2},\ldots ,synth\_sc_{64}\right] \end{array}$$

**Figure 6:**
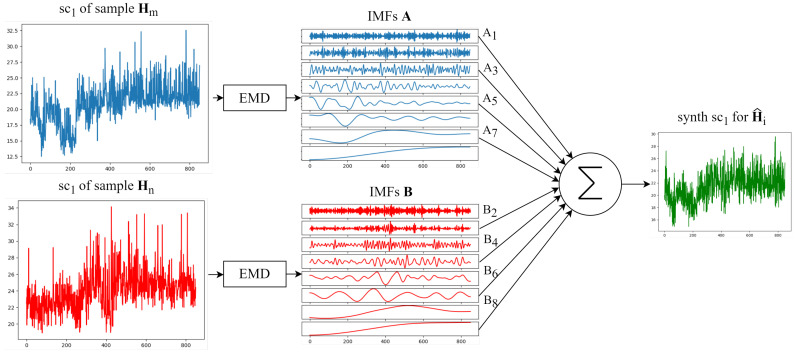
Process for generating a synthetic subcarrier from the same subcarrier index of two different fall samples.

The process of creating a synthetic subcarrier signal from two different samples of the same individual is illustrated in Figure 6. The complete process for creating the synthetic dataset $\hat{H}$ used in this work is presented in Algorithm 1.
**Algorithm 1:** EMD-based data augmentation
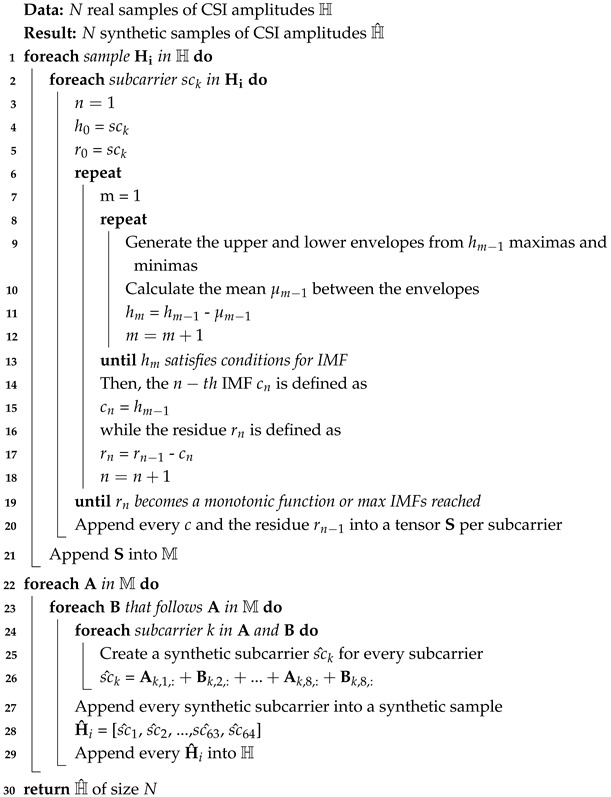


**Figure 7:**
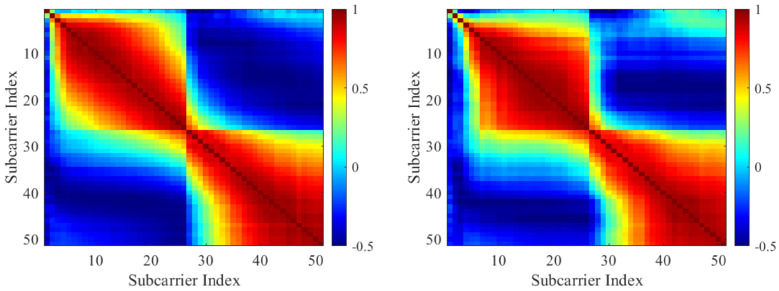
Correlation between subcarriers. **Left**: correlation between subcarriers from a real fall sample. **Right**: correlation between subcarriers from a synthetic sample.

Wi-Fi adjacent subcarriers exhibit inherent correlation as a result of multipath propagation and the constraints imposed by coherence bandwidth [30]. As illustrated in Figure 7, this correlation observed among real subcarrier samples is similarly present in the synthetic samples. This suggests that the synthetic data effectively replicates the statistical behavior of a real-world wireless channel.

## 5. The DenseNet-Based HAR Model with Wi-Fi CSI

DenseNet proposes direct connections from any layer to subsequent layers within structures called Dense Blocks. This dense connectivity pattern helps to mitigate the vanishing gradient and inherently strengthens feature propagation throughout the network. Each Dense Block consists of *n* sequences of a composite function comprising Batch Normalization, a Rectified Linear Unit, and a 3×3 Convolutional Layer, which will be referred to as the Main Convolutional Layer. Additionally, a Bottleneck Layer is placed before the Main Convolutional Layer to reduce the number of input feature maps, thereby decreasing computational complexity. Within the Dense Block, the number of feature maps added by each Main Convolutional Layer is determined by a parameter called the Growth Rate, which controls how much new information each layer contributes to the global state [31].

**Figure 8:**
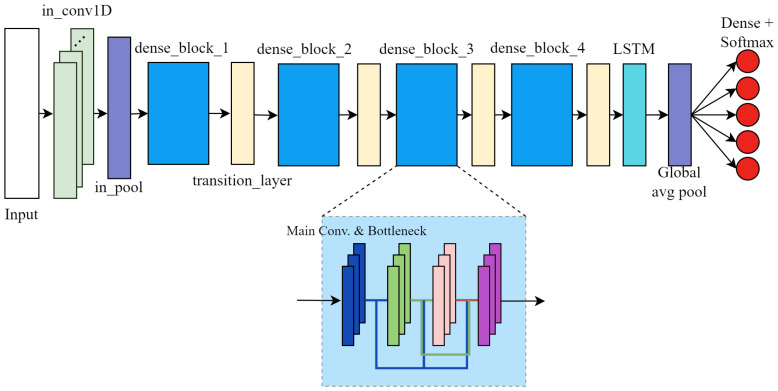
LSTM-embedded DenseNet model architecture.

The HAR model utilizing CSI, as proposed in this study, is based on the DenseNet architecture, with key modifications introduced to accommodate the specific characteristics of the collected data. First, the conventional 2D convolutional layers typically effective for spatial feature extraction in image data are replaced with 1D convolutions. This adjustment is essential as CSI data inherently represents time series information across individual subcarriers. Second, a LSTM layer is incorporated following the convolutional blocks to capture the temporal features of human activity reflected in the amplitude variations of CSI subcarriers. This modified architecture, referred to as the LSTM-Embedded DenseNet model, is illustrated in Figure 8. The feature maps produced by the final Dense Block are fed into the LSTM layer, which processes the sequence to generate a context-rich encoded representation for the classification layer, consisting of a dense layer with the Softmax activation function.

It is worth emphasizing that given the model’s intended deployment on embedded devices, the number of layer sequences within each Dense Block is substantially reduced compared to the standard DenseNet121 architecture. This optimization strategy is designed to minimize inference latency and reduce model size while preserving competitive performance, thereby ensuring suitability for real-time applications on resource-constrained edge platforms. A detailed breakdown of the model’s architecture including the specific number of layers, growth rate, and feature map dimensions at each stage is provided in Table A1.

## 6. Materials and Methods

### 6.1. Data Collection

To construct the datasets used for training and evaluating the HAR models, the ESP32 CSI web collecting tool was flashed on two Espressif ESP32-DevkitCVIE development boards, one configured as Tx and the other as Rx. Each board was equipped with an Espressif ESP32-D0WD-V3 dual-core processor operating at 240 MHz, alongside 8 MB of flash memory and 8 MB of PSRAM. The tool was configured to operate over a 20 MHz Wi-Fi channel, transmitting and receiving UDP packets at a rate of 50 packets per second.

On the Rx side, the tool was configured in *Console Mode*, which streamed CSI estimations in binary format via the device’s USB port. Additionally, a Python 3.11 script running on an HP ProDesk 400 G6 Desktop Mini PC was developed to capture and store these estimations in comma-separated files, where each file represented a single sample.

A total of 22 volunteers participated in this study, each performing 15 repetitions of five distinct activities, with each repetition lasting 20 s. The activities were defined as follows:

- Lie Down (LD): The volunteer began standing at point $A_{p}$. After five seconds, the volunteer sat on a camping cot and transitioned to a lying position.
- Get Up (GU): Starting from a lying position on the camping cot, the volunteer remained stationary for five seconds before rising to a standing position at point $A_{p}$.
- Sit Down (SD): The volunteer stood behind a chair placed at point $A_{p}$. After five seconds, the volunteer walked around the chair and sat down.
- Fall (FA): The volunteer stood adjacent to a floor-level mattress at $A_{p}$. After five seconds, the volunteer lay on the mattress at a fast pace, simulating a fall.
- Walk (WA): The volunteer walked continuously between points $WP_{1}$ and $WP_{2}$ for the duration of the recording.

**Figure 9:**
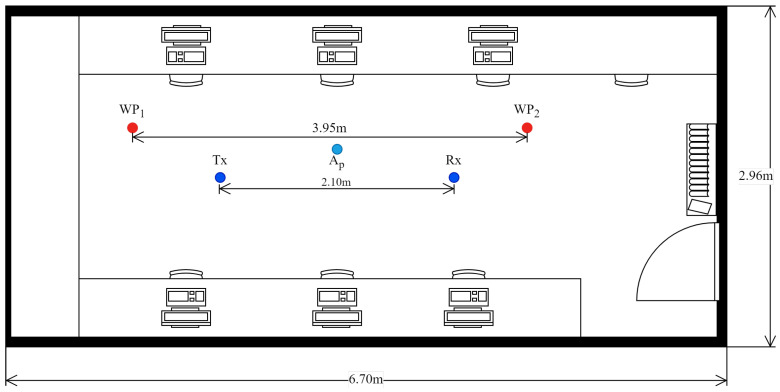
CSI collection scenario. $WP_{1}$ and $WP_{2}$ are the reference points for walking. $A_{p}$ is the reference point for fall, sit down, lie down, and get up activities.

All experimental activities were conducted within a computer laboratory, with only the volunteer present during each session (see Figure 9). The laboratory was situated in a building surrounded by other rooms and laboratories occupied by individuals and equipped with multiple Wi-Fi devices operating on the 2.4 GHz band. Consequently, the data collection occurred in a quasi-realistic environment characterized by external interference while maintaining a controlled setting with a single participant inside the room. Prior to participation, each volunteer provided informed consent, authorizing the use of the collected data for academic research purposes.

A total of 1647 samples were collected, which were further divided into training and test sets. Table 2 shows the frequencies distribution for both datasets.

### 6.2. Data Preprocessing

The collected samples comprised complex-valued CSI estimation, with each estimation represented as a complex pair for every subcarrier. From these complex pairs, the CSI amplitude was computed using the following equation:

(7) $$\begin{array}{c} \left|H\right|=\sqrt{Re^{2}+Im^{2}} \end{array}$$

As a result, 64 amplitude values were calculated for each CSI estimation. However, this number was later reduced to 52 by excluding those identified as null and pilot subcarriers, as well as subcarriers identified as problematic based on empirical experimentation. Furthermore, only 47 of the 52 were retained for further experimentation.

The CSI amplitude data were organized into NumPy arrays to facilitate efficient processing using Python scripts and TensorFlow 2.18. Although the acquisition tool was configured to capture 50 packets per second over a 20-second interval—yielding a theoretical total of 1000 timesteps—each sample was standardized to 850 timesteps to ensure consistent array dimensions across the dataset. This adjustment compensated for variability in packet reception rates and fluctuations in the actual number of packets received at the receiver (Rx) side.

As previously noted, the volunteer was the sole occupant of the computer lab during data collection, situated within a typical office environment. Ambient factors included nearby Wi-Fi devices operating on the same frequency band, which introduced interference and contributed to packet loss.

The EMD-based data augmentation algorithm was used to increase the number of samples for training, thus constructing a second training set consisting of real and synthetic samples arranged into a numpy array. By using this algorithm, it was possible to expand the number of samples from 1198 in the training set to 11,513 samples.

### 6.3. Model Training and Evaluation

Model training was performed on a desktop computer equipped with an Intel Core i5-12400F processor, 48 GB of RAM, and an NVIDIA GeForce RTX 4060 Ti GPU featuring 8 GB of VRAM. Three distinct models were developed for comparative evaluation.

1. The first model served as a baseline and was trained exclusively on the original training set, which comprised only real samples. Although it incorporated an LSTM layer with 128 units, the number of sequences in each Dense Block was aligned with the DenseNet121 architecture.
2. The second model retained the same architectural framework but was trained on an augmented dataset to assess the impact of data augmentation on performance.
3. The third model introduced the proposed LSTM-embedded DenseNet architecture and was trained using the augmented dataset, which included both real and synthetically generated samples. This final model was subsequently deployed to an embedded device following quantization via TensorFlow Lite tools.

Each model was trained over 300 epochs with a batch size of 32 and a learning rate of 0.0001, utilizing the Adam optimizer for gradient-based optimization. Model performance was assessed using a separate test set composed of samples excluded from the training phase. Evaluation metrics included average accuracy, precision, and recall, computed across all five target classes to ensure a comprehensive performance comparison.

An ESP32-S3 N16R8 development board, featuring 16 MB of flash memory and 8 MB of external PSRAM, was employed to deploy the quantized LSTM-embedded DenseNet model and perform classification on each sample in the test set.

The quantization process involved converting the model’s inputs, outputs, and weights from 32-bit floating-point representations to 8-bit integer values [12]. This transformation significantly reduced both model size and inference latency, as integer operations are computationally more efficient than their floating-point counterparts, albeit with a minor trade-off in accuracy.

In addition to the previously reported performance metrics, two deployment-specific parameters were measured: model latency, defined as the time elapsed between receiving an input and generating an output, and memory footprint, referring to the amount of memory required to allocate the quantized model on the embedded device.

## 7. Results and Discussion

### 7.1. Performance Evaluation of the CSI Collecting Tool

To identify the maximum sustainable packet transmission rate that preserved reliable CSI estimation at the Rx, an initial evaluation was conducted by transmitting UDP packets at rates ranging from 20 to 200 packets per second. For each transmission rate, the number of successfully received packets at the Rx was recorded over a 20-min interval.

To isolate potential bottlenecks caused by secondary processes—such as CSI reporting to an external device or SD card storage—the informer task at the Rx was disabled during this evaluation. This ensured that the observed performance metrics reflected the transmission and reception capabilities without interference from auxiliary operations.

The results of this evaluation are presented in Table 3. As Rx performed CSI estimation on a per-packet basis, it was observed that not all transmitted packets were successfully received. This phenomenon can be attributed to the inherent unreliability of the UDP protocol, which did not guarantee packet delivery, and the interference from other Wi-Fi devices operating in the same frequency band.

Packet rates above 200 were not supported as the task watchdog was triggered, indicating system overload. The measured inter-packet intervals provided critical insight into the real-time processing requirements for CSI measurements. For instance, at the maximum supported rate of 200 packets per second, the complete CSI estimation and processing pipeline needed to be executed within 5 ms per sample to prevent system failures due to computational bottlenecks.

The second evaluation assessed the maximum sustainable packet rate while ensuring reliable transmission of CSI estimations through both the USB-to-UART serial interface and SD card storage. Since serial communication transmits data sequentially (bit by bit), the baud rate fundamentally constrains the maximum achievable packet rate for CSI data transmission via UART interfaces.

Theoretically, the packet rate, in function of the baud rate, is given by the following equation:

(8) $$packet\;rate=\frac{baudrate}{frame\;size}$$

When using ASCII encoding, each digit of a CSI subcarrier measurement—covering both real and imaginary components—requires 8 bits for representation. Given the ESP32 Wi-Fi API’s 8-bit resolution, which spanned values from −128 to 127, the worst-case scenario involved representing each measurement as a three-digit negative value. Under these conditions, a single subcarrier could require up to 64 bits. Consequently, a full CSI measurement comprising 64 subcarriers could demand approximately 4096 bits.

If subcarrier measurements are comma-separated, the total frame size increases to roughly 5112 bits. By substituting this frame size and the configured baud rate of 230,400 bps into Equation (8), the maximum achievable packet transmission rate was estimated at 45 packets per second.

Alternatively, if the format is set to binary—preserving CSI values directly as 8-bit integers without ASCII encoding—the frame size for 64 subcarriers reduces to 1024 bits. This optimization yields a theoretical maximum transmission rate of approximately 225 packets per second.

To empirically validate the theoretical transmission limits, an ESP32 was configured as Tx to send UDP packets at rates ranging from 10 to 100 packets per second, incremented in steps of 10. A second ESP32, configured as Rx, operated in Console Mode, enabling CSI estimations to be transmitted via USB to a host computer running a Python script. This script recorded the average number of CSI estimations received per second over a 20-min interval at a baud rate of 230,400 bps. Both Tx and Rx devices were ESP32-DevkitCVIE development boards, each operating at a clock frequency of 240 MHz and equipped with 8 MB of flash memory and 8 MB of external PSRAM.

**Figure 10:**
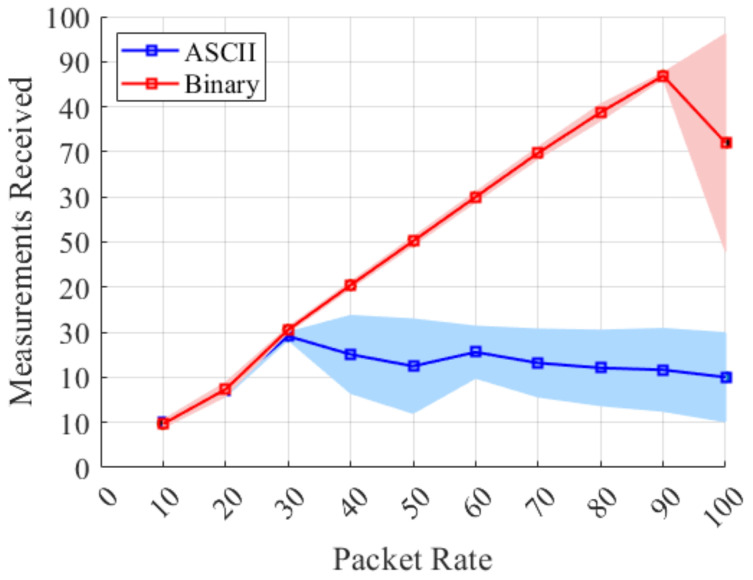
CSI measurements received in the computer at different Tx–Rx packet rates. Standard deviation is illustrated by the shaded area.

The results presented in Figure 10 reveal a strong correspondence between theoretical predictions and empirical measurements for ASCII-formatted CSI data at transmission rates below 30 packets per second. Beyond this threshold, the rate of successfully received measurements plateaud at approximately 30 pps, indicating saturation of the serial communication channel. This behavior not only validates the theoretical calculations but also highlights practical constraints in maintaining sustained data throughput under ASCII encoding.

Notably, when the sample format was set to binary, the maximum achievable packet rate fell significantly below—and was less than half of—the theoretical limit of 225 packets per second. While the average number of CSI measurements received by the host computer initially aligned with the configured transmission rate, this correspondence diminished beyond 80 packets per second. The number of received measurements stabilized at around 85 packets per second.

This discrepancy was primarily attributed to the execution time of the write instruction used to transmit data via the board’s USB interface, which exceeded the inter-arrival time of incoming UDP packets. A similar bottleneck was observed when CSI measurements were stored directly on an SD card, indicating that I/O latency imposes a practical constraint on sustained data throughput.

### 7.2. Model Evaluation Results

The evaluation of baseline LSTM-DenseNet121 models provided important insights into the impact of data augmentation on the performance of HAR models. The first models evaluated, which served as benchmarks for those quantized and implemented on the ESP32, included the LSTM-DenseNet121 model, both with and without data augmentation.

**Figure 11:**
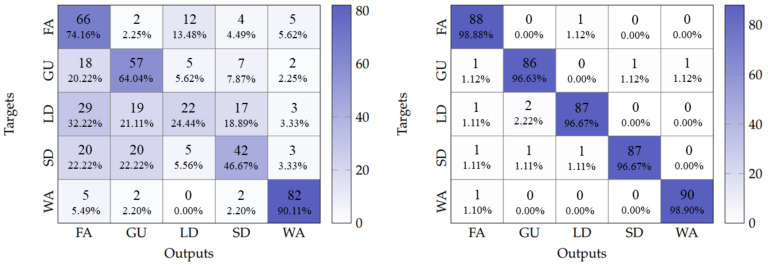
Confusion matrix for HAR with LSTM-DenseNet121. **Left**: no data augmentation. **Right**: with data augmentation.

When tested, the baseline model with no data augmentation achieved an overall accuracy of 59.91%. In contrast, the model with data augmentation demonstrated a significant improvement, reaching an overall accuracy of 97.55%. Figure 11 shows the confusion matrix obtained by evaluating these two models. The non-augmented model exhibited significant missclassifications between LD and FA classes, which was likely due to the similar movement patterns associated with both activities, differing primarily in execution pace. However, this situation was completely resolved in the augmented model.

These results indicate that data augmentation allows the LSTM-DenseNet121 architecture to learn robust temporal features, effectively differentiating between kinematically similar activities that vary mainly in speed, thereby achieving reliable HAR.

**Figure 12:**
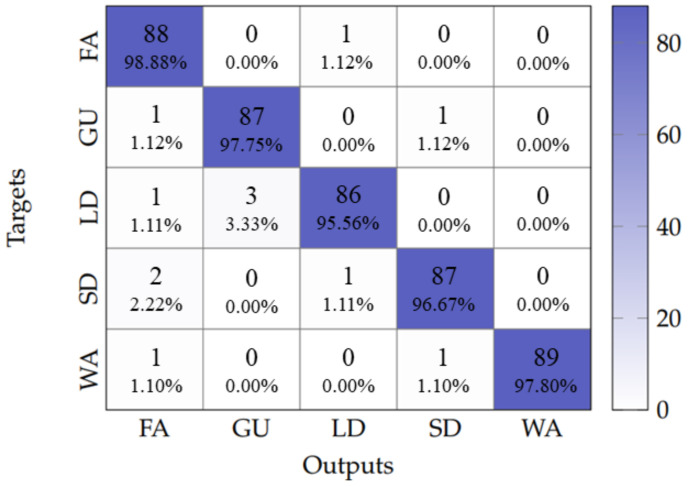
Confusion matrix obtained for the LSTM-embedded DenseNet model.

Having established this performance baseline, it was possible to analyze whether architectural simplification for embedded deployment affected model accuracy. The LSTM-Embedded DenseNet model achieved 97.33% accuracy, only 0.21% lower than the LSTM-DenseNet121, while reducing parameters from 437,234 to 122,383, which gave a 72% reduction with negligible accuracy impact (see Figure 12). This suggests that the optimized architecture successfully maintains the baseline’s temporal discrimination capabilities while achieving substantial efficiency gains for embedded implementation.

The previously stated results were obtained by executing the model on a computer. To implement the LSTM-embedded DenseNet model on the ESP32-S3, a full unsigned 8-bit integer quantization of its inputs, outputs, and weights was performed using the TensorFlow Lite framework. This was followed by deployment to the device using TensorFlow Lite Micro for Espressif Chipsets.

After quantization and deployment, the overall accuracy obtained by the model decreased to 93.32%; however, model quantization is a mandatory step for deploying a model into any microcontroller for optimal performance. On the other hand, considering that a single sample consisted of multiple subcarriers, the model latency was 10,904 ms, i.e., almost 11 s. This can be considered far from near-real-time functioning, as a person can perform more than one activity in that time.

**Figure 13:**
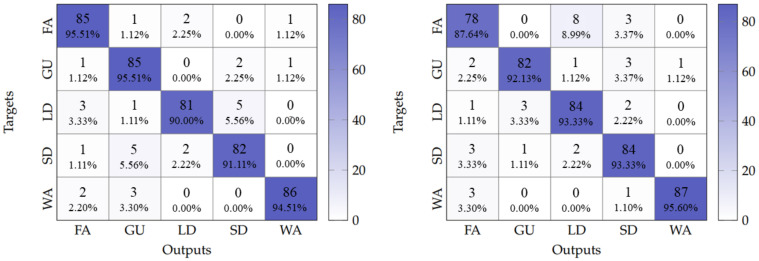
Confusion matrix obtained for the embedded model with full 8-bit quantization. **Left**: with every subcarrier. **Right**: most sensitive subcarrier only.

Hence, a subcarrier selection method based on variance [32] was used for selecting the subcarrier that best reflected a human movement. This method was chosen for its simplicity and effectiveness. It involved calculating the variance for each subcarrier in a sample and selecting the *n* subcarriers with the highest variance values. The experimental results indicate that subcarriers with higher variance values are more likely to contain relevant information for Wi-Fi sensing applications [32,33]. By selecting a single subcarrier using this method, an overall accuracy of 92.43% was obtained, while the model latency was reduced to only 232 ms. Figure 13 presents the confusion matrices obtained for these two cases.

In addition, the LSTM-DenseNet121 model was also quantized and implemented into the ESP32-S3 by performing single subcarrier classification, achieving an overall accuracy of 95.55%. However, due to the greater number of parameters because of Dense Block layers, the model latency was increased to 817 ms. This increase was also reflected in the amount of memory allocated for the model, which was 277 kB for the LSTM-DenseNet121, while, for the LSTM-embedded DenseNet model, only 127 kB needed to be allocated. Consequently, having an external PSRAM is a must. All reported results related to models’ performance are summarized in Table 4.

The results highlight a trade-off between model depth, accuracy, latency, and memory usage. While increasing depth—particularly by adding additional layers to the Dense Blocks—classification accuracy can be improved; it also incurs higher latency and memory consumption due to the increased number of parameters and computational operations the device must handle.

A practical strategy to mitigate these resource demands is model quantization, which involves converting a model’s inputs, outputs, and weights from 32-bit floating-point values to 8-bit integers. This transformation significantly reduces memory requirements and accelerates inference, as integer operations are inherently more efficient than floating-point computations. However, this optimization comes at a modest cost. A 4% reduction in accuracy was observed for the LSTM-embedded DenseNet model following quantization.

## 8. Conclusions and Future Work

This work introduced the ESP32 CSI web collecting tool, a flexible and user-friendly solution for CSI data acquisition. Unlike conventional tools, it enables configuration through a web interface, eliminating the need for source code modifications or compilation parameter adjustments within the development framework. This design facilitates in-field reconfiguration, allowing researchers to modify communication parameters or adapt the device to evolving experimental requirements without physically relocating it.

The tool supports baud rate adjustments to achieve higher packet transmission rates; however, compatibility with the connected device must be considered, particularly in relation to the UART crystal oscillator frequency.

Empirical evaluations demonstrate that the proposed tool outperforms existing CSI collection solutions when deployed on ESP32-based platforms. Its ability to sustain higher packet rates makes it especially suitable for demanding Wi-Fi sensing applications, where throughput and responsiveness are critical.

In parallel, the proposed EMD-based data augmentation algorithm demonstrated its effectiveness in improving the performance of Wi-Fi CSI-based HAR models. Specifically, it elevated the accuracy of the LSTM-DenseNet121 architecture from 59.91% to 97.54%, underscoring the quality and relevance of the synthetic samples generated. This substantial improvement highlights that the augmented data successfully preserves the intrinsic characteristics of the Wi-Fi channel—particularly the correlation between adjacent subcarriers—thereby maintaining the statistical integrity required for robust model training.

Taking into account the hardware limitations of the ESP32 platform, this study successfully optimized DenseNet-based models to achieve near-real-time performance with reduced memory consumption while maintaining satisfactory classification accuracy. The LSTM-embedded DenseNet model attained an overall accuracy of 92.43% using single subcarrier selection for the recognition of five distinct activities. Notably, an accuracy of 95.55% was achieved under optimal conditions, aligning with state-of-the-art results reported in the related literature.

However, this gain in performance comes at the cost of increased computational complexity, as reflected in elevated model latency and memory usage. When near-real-time operation is required, it is crucial to recognize that deeper architectures inherently introduce latency due to the greater number of operations involved.

To address this challenge, one viable solution is the deployment of AI-specialized microcontrollers—such as those in the STM32N6 series—which incorporate Neural Processing Units (NPUs) designed for efficient edge AI inference. These devices offer enhanced computational capabilities, enabling the execution of deeper models with reduced latency and improved energy efficiency.

While CSI collection and HAR have been successfully implemented on ESP32 devices, future enhancements to the proposed tool include integrating Octa-SPI communication with Direct Memory Access (DMA) to enable efficient device-to-device transmission of CSI data. A dual-microcontroller configuration is recommended: one ESP32 dedicated to CSI acquisition and the other functioning as an NPU for HAR inference.

The current implementation relies on read/write cycles that consume CPU time, introducing latency in inter-device communication. Although this overhead falls outside the scope of the present study, future integration of DMA is expected to significantly reduce CPU load and improve data transfer efficiency. Accordingly, future work will focus on designing a dedicated development board for Wi-Fi sensing applications that consolidates data acquisition and model execution within a single, optimized hardware platform.

It is also important to acknowledge the limitations of the current experimental setup. Although CSI data were collected in the presence of ambient Wi-Fi activity and surrounding individuals, the volunteer was alone in the room during activity execution, and all actions were performed in a consistent manner. No cross-domain HAR or multi-person recognition experiments were conducted. These scenarios represent more complex environments, where model performance may vary.

Therefore, future research will aim to extend HAR capabilities to cross-domain and multi-user contexts under dynamic conditions, incorporating a broader range of activities. The ultimate goal is to achieve robust, real-time recognition using only embedded devices, even in challenging and variable environments.

## Figures and Tables

**Table 1:** Comparison of Wi-Fi CSI collecting tools.

Tool	Device	CSI Resolution	N^o^ Subcarriers	802.11 Support	Configuration
Linux 802.11n CSI Tool [13]	Intel 5300 NIC	8-bit	Grouping of 30	a/b/g/n	Through Linux commands
Atheros CSI Tool [14]	Atheros chips	10-bit	56 and 114 for 20 MHz and 40 MHz channels	a/b/g/n	Through Linux commands
Nexmon [15,16]	Broadcom and Cypress Wi-Fi chips	Not specified	256 and 2048 for 80 MHz and 160 MHz channels	a/b/g/n/ac/ax	Through Linux commands
ESP32 CSI Tool [17]	ESP32 devices	8-bit	64 and 114 for 20 MHz and 40 MHz channels	a/b/g/n	Through source code/framework configuration

**Table 2:** Activity distribution in training and test sets.

Activity	Training Set	Test Set	Total
LD	239	90	329
GU	240	89	329
SD	242	90	332
FA	238	89	327
WA	239	91	330

**Table 3:** Packet rate evaluation results.

Configured Packet Rate	Rx CSI Estimations	Interval Between Packets (ms)
20	18.7302	50
40	39.9134	25
60	59.3816	16.67
80	77.8126	12.5
100	97.8766	10
120	115.0033	8.33
140	136.4282	7.14
160	148.4420	6.25
180	163.8078	5.55
200	182.0546	5

**Table 4:** Performance results for HAR models.

	LSTM-Embedded DenseNet			LSTM-DenseNet121	
	TF fp32 Model ^1^	Quant. Model	Quant. Model with ss ^2^	TF fp32 Model ^1^	Quant. Model with ss ^2^
Accuracy	97.33%	93.32%	92.43%	97.55%	95.55%
Precision	97.35%	93.40%	92.52%	97.56%	95.64%
Recall	97.33%	93.33%	92.41%	97.55%	95.54%
Latency (ms)	-	10,904	232	-	817
Size (kB)	-	127	127	-	277

^1^ Model running in personal computer without quantization, only for reference purposes. ^2^ Subcarrier selection.

## Data Availability

The original data and scripts presented in this study are openly available in a GitHub repository at https://github.com/AlbanyArmenta0711/Embedded_WiFi_Sensing, accessed on 2 October 2025.

## Abbreviations

The following abbreviations are used in this manuscript:

CSI	Channel State Information
HAR	Human Activity Recognition
LoS	Line-of-Sight
PHY	Network Physical Layer
LTF	Long Training Field
OFDM	Orthogonal Frequency Division Multiplexing
NIC	Network Card Interface
Rx	Receiver
AP	Access Point
Tx	Transmitter
NVS	Non-Volatile Storage
EMD	Empirical Mode Decomposition
IMF	Intrinsinc Mode Function
LD	Lie Down
GU	Get Up
SD	Sit Down
FA	Fall
WA	Walk
DMA	Direct Memory Access
NPU	Neural Processing Unit

## Appendix B. LSTM-Embedded DenseNet Architecture

**Table A1:** Description of each model block and layer for LSTM-embedded DenseNet. The growth rate of the network was set to 11 and the compression factor was set to 0.5.

Layers	Output Size	Details
Input	850×1	Single CSI subcarrier
Input Convolution	425×11	kernel size = 7, stride = 2
Max Pooling	212×11	pool size = 3, stride = 2
Dense Block 1	212×44	Bottleneck ×3
		kernel size = 1, stride = 1
		Main Conv. ×3
		kernel size = 3, stride = 1
Transition Layer 1	215×5	Conv. Layer
		kernel size = 1, stride = 1
	106×5	Avg. Pooling
		pool size = 2, stride = 2
Dense Block 2	106×71	Bottleneck ×6
		kernel size = 1, stride = 1
		Main Conv. ×6
		kernel size = 3, stride = 1
Transition Layer 2	106×5	Conv. Layer
		kernel size = 1, stride = 1
	53×5	Avg. Pooling
		pool size = 2, stride = 2
Dense Block 3	53×137	Bottleneck ×12
		kernel size = 1, stride = 1
		Main Conv. ×12
		kernel size = 3, stride = 1
Transition Layer 3	53×5	Conv. Layer
		kernel size = 1, stride = 1
	26×5	Avg. Pooling
		pool size = 2, stride = 2
Dense Block 4	26×93	Bottleneck ×8
		kernel size = 1, stride = 1
		Main Conv. ×8
		kernel size = 3, stride = 1
Transition Layer 4	26×5	Conv. Layer
		kernel size = 1, stride = 1
	13×5	Avg. Pooling
		pool size = 2, stride = 2
LSTM	13×32	units = 32
Classification Layer	1×5	Global Avg. Pooling
		Fully Connected + Softmax
